# ChIP-Seq Analysis of the σ^E^ Regulon of *Salmonella enterica* Serovar Typhimurium Reveals New Genes Implicated in Heat Shock and Oxidative Stress Response

**DOI:** 10.1371/journal.pone.0138466

**Published:** 2015-09-21

**Authors:** Jie Li, Christopher C. Overall, Rudd C. Johnson, Marcus B. Jones, Jason E. McDermott, Fred Heffron, Joshua N. Adkins, Eric D. Cambronne

**Affiliations:** 1 Department of Molecular Microbiology and Immunology, Oregon Health & Science University, Portland, Oregon, United States of America; 2 Biological Sciences Division, Pacific Northwest National Laboratory, Richland, Washington, United States of America; 3 Department of Infectious Diseases, J. Craig Venter Institute, Rockville, Maryland, United States of America; University of Osnabrueck, GERMANY

## Abstract

The alternative sigma factor σ^E^ functions to maintain bacterial homeostasis and membrane integrity in response to extracytoplasmic stress by regulating thousands of genes both directly and indirectly. The transcriptional regulatory network governed by σ^E^ in *Salmonella* and *E*. *coli* has been examined using microarray, however a genome-wide analysis of σ^E^–binding sites in *Salmonella* has not yet been reported. We infected macrophages with *Salmonella* Typhimurium over a select time course. Using chromatin immunoprecipitation followed by high-throughput DNA sequencing (ChIP-seq), 31 σ^E^–binding sites were identified. Seventeen sites were new, which included outer membrane proteins, a quorum-sensing protein, a cell division factor, and a signal transduction modulator. The consensus sequence identified for σ^E^ *in vivo* binding was similar to the one previously reported, except for a conserved G and A between the -35 and -10 regions. One third of the σ^E^–binding sites did not contain the consensus sequence, suggesting there may be alternative mechanisms by which σ^E^ modulates transcription. By dissecting direct and indirect modes of σ^E^-mediated regulation, we found that σ^E^ activates gene expression through recognition of both canonical and reversed consensus sequence. New σ^E^ regulated genes (*greA*, *luxS*, *ompA* and *ompX*) are shown to be involved in heat shock and oxidative stress responses.

## Introduction

Where household sigma factor σ^70^ is responsible for promoting transcription of a large number of genes in a bacterial cell, alternative sigma factors are produced or activated when cells undergo particular physiological stresses [1] [2]. The alternative sigma factor E (σ^E^) responds to extracytoplasmic cues generated by temperature, osmotic, or oxidative stress, which releases σ^E^ from sequestration at the inner membrane through a series of protein cleavage events. Free σ^E^ in the cytoplasm binds core RNA polymerase and initiates transcription at σ^E^-dependent promoters, resulting in a specific response to promote restoration of homeostasis in the cell [3] [4]. In *Salmonella enterica* serovar Typhimurium (referred to as STM or *Salmonella* hereafter), deletion of σ^E^ (*ΔrpoE*) is lethal for intracellular survival in macrophages; the strongest phenotype among regulator deletion strains examined [5].

The transcriptional regulatory network controlled by σ^E^ has been investigated in previous studies [6] [7] [8] [9]. Recent findings from our group indicated that approximately 58% of the entire *Salmonella* genome was regulated by σ^E^, an effect most likely produced by modulation of the expression of multiple general regulators [10]. Aided by sample-matched global proteomic and transcriptomic analyses, we found that σ^E^ regulated *Salmonella* gene expression not only at the transcriptional level, but also by a post-transcriptional mechanism, which was partially dependent on the RNA-binding protein Hfq [11]. The above studies suggested that the majority of transcriptional regulation mediated by σ^E^ occurred indirectly, driven by a small number of genes that were directly regulated by σ^E^. Therefore, it is essential to identify σ^E^-binding sites globally to understand the intricacies of σ^E^-mediated gene regulation.

Chromatin-immunoprecipitation followed by high-throughput DNA sequencing (ChIP-seq) has been successfully used in the identification of chromosomal binding sites of various regulators in bacteria [12] [13] [14]. It offers higher resolution, lower noise, and greater coverage than its array-based predecessor ChIP-chip [15]. We selected ChIP-seq as a method for genome-wide profiling of σ^E^–binding sites in STM. Our results indicated some σ^E^–binding sites resided 5’ to divergent flanking genes, and raised the question whether σ^E^ regulated these genes equally. Moreover, a previous study from the Gross group mentioned the possibility that a reversed consensus sequence of σ^E^ might be involved in repressing gene expression, exemplified by *ompX* [8]. We elucidated the above questions by constructing consensus sequence substituted strains, and dissecting direct from indirect regulatory effects of σ^E^.

In this study we identified 31 σ^E^–binding sites on the STM genome during *in vivo* infection of Raw264.7 macrophages with STM and determined a consensus sequence for σ^E^-binding. However, this consensus sequence was not the only mechanism utilized by σ^E^ in transcriptional regulation. Moreover, σ^E^ did not regulate its flanking genes equally when the σ^E^-binding site resided between bi-directional promoters. σ^E^ activated gene expression through binding the consensus or reversed consensus sequence. Finally, we found that new targets directly regulated by σ^E^ were involved in response to heat shock and oxidative stress.

## Materials and Methods

### Bacterial strains and growth conditions

*Salmonella enterica* Serovar Typhimurium ATCC 14028s was used as the parent strain in this study. The *rpoE*-deletion strain (*ΔrpoE*) was constructed using λ red recombination system as described [5]. The consensus sequence substituted mutants were constructed by replacing the σ^E^–binding motif of *greA*, *ompX*, *ompA*, *luxS* and *rpoE* with a substitutive consensus sequence consisting of nucleotides with the least prevalence at the corresponding positions in the σ^E^ consensus sequence (ATTTGCGGGAACATGCGAAGACTGACTG). The substitutive consensus sequence was synthesized with *SacI* and *AvrII* restriction sites at two ends, and ligated into pKD13 modified plasmid [16], resulting in a new plasmid pKD13-RpoE1. λ red recombination was used to construct the consensus sequence substituted mutants with pKD13-RpoE1 plasmid similar to the *ΔrpoE* strain. All substitutions were validated by DNA sequencing. Primers used for construction of the above mutants are shown in S1 Table.

For *in vitro* study, the bacteria were grown overnight in Luria-Bertani (LB) medium, washed twice in pH 5.8, low phosphate, low magnesium-containing medium (LPM), and resuspended in LPM at 1:10 dilution for an additional 4 h, 8 h or 20 h [17]. All bacterial cultures were grown in triplicate.

### Anti-σ^E^ antibody generation

The anti-σ^E^ antibody (Ab) was generated as previously described [10]. Briefly, the *rpoE* gene of STM was cloned into the plasmid pET200/D-TOPO (Invitrogen) and transformed into BL21 (DE3) *E*. *coli* strain (Invitrogen). Transformed bacteria were induced using Isopropyl β-D-1-thiogalactopyranoside (IPTG), lysed and sonicated for purification of recombinant σ^E^ using HisPur^TM^ Cobalt resin (Pierce). The eluted protein was separated on SDS-PAGE, and stained with Coomassie blue. The single gel bands at the size of recombinant RpoE (His tagged) were excised and sent to Pacific Immunology Corp. (Ramona, CA) for polyclonal antisera production. The σ^E^ antisera generated in rabbits were purified by affinity chromatography. CH Sepharose 4B (GE Healthcare) was coupled with recombinant σ^E^, then σ^E^ antisera was loaded in a chromatography column (Bio-Rad). After washes, the column was eluted with 200 mM glycine pH 2.0, then adjusted to pH 7.0 with 5 N NaOH. Fractions containing purified anti-σ^E^ Ab as judged by SDS-PAGE were frozen at -20°C. Protein concentration determination was performed according to modified Lowry method using bovine serum albumin (BSA) as reference protein [18].

### Immunoblot analysis

The WT and *ΔrpoE* strains were cultured in LB for 4 h. Cells were washed and approximately 5 x 10^7^ colony-forming units were pelleted and re-suspended in Laemmli sample buffer, boiled for 5 min, and then separated on SDS-PAGE. Proteins on the gel were next transferred to polyvinylidene difluoride (PVDF) membrane (Millipore). After blocking in Tris-buffered saline (TBS) plus 5% nonfat dry milk for 1 h, membranes were probed with anti-σ^E^ Ab or anti-GFP Ab at 1:1000, 1:3000, and 1:5000 dilutions. The flow-through of rabbit immune sera during affinity chromatography purification of anti-σ^E^ Ab was also used to probe the membrane as control. Membranes were washed and probed with secondary antibody (anti-rabbit IgG conjugated with peroxidase) (Cell Signaling Technology). The immune complexes were detected via chemiluminescence using Western Lightning^TM^ (PerkinElmer), and then exposed to XAR Biofilm (Kodak).

### *Salmonella* infection

The Raw264.7 macrophage cell line was purchased from ATCC and grown in Dulbecco’s modified Eagle’s medium (DMEM) containing 10% fetal calf serum in 6- well plates until reaching confluency of 80–90%. STM grown overnight in LB was washed and diluted with PBS to an appropriate concentration. Raw264.7 cells were infected with STM at MOI = 100 for 4, 8, or 18 h. Infections were initiated by centrifuging the bacteria onto the cell monolayers at 1,000 × *g* for 5 min and plates were incubated at 37°C with 5% CO_2_ for 1 h. To remove extracellular bacteria after internalization, cells were washed with PBS and incubated in DMEM containing 10% fetal calf serum and gentamicin (100 μg/ml) for 1 h. The cells were then washed with PBS and overlaid with DMEM containing 10% fetal calf serum and 20 μg/ml gentamicin for the remainder of the experiment.

### Chromatin Immunoprecipitation followed by sequencing (ChIP-seq)

After 4, 8, or 18 h infection with STM, Raw264.7 cells were washed with PBS and scraped off the plates. The cells were collected and cross-linked by 1% formaldehyde at room temperature for 25 min with shaking, then quenched using 125 mM glycine for an additional 5 min of incubation at room temperature. Chromatin Immunoprecipitation assay was performed as described [10]. Cells were lysed and sonicated, and the supernatant was split into two samples. One was mixed with affinity purified rabbit anti-σ^E^ Ab to immunoprecipitate σ^E^–DNA complex, and the other sample was mixed with rabbit monoclonal Ab to GFP as control. After overnight incubation at 4°C, 50 μl of the Dynabead M-280 sheep anti-rabbit IgG (Invitrogen) was added into the mixture and further incubated at 4°C for 6 h. Beads were washed and resuspended in elution buffer (50 mM Tris-HCl at pH 8.0, 10 mM EDTA, and 1% SDS) and incubated at 65°C overnight to reverse the cross-linking. Protein and RNA were removed from the samples and DNA was purified with a PCR purification kit (QIAGEN). Gene-specific quantitative PCR was carried out using the DNA samples (primers in S2 Table).

The DNA was then combined with 5x Sequenase buffer and Random 9-Ns primer and cycled to 94°C for 2 min then cooled to 10°C. A mix of 5x Sequenase buffer, dNTP mix, BSA, DTT, and Sequenase was added to the DNA and ramped up from 10°C to 37°C at 0.1°C/s, held at 37°C for 8 min, heated to 94°C for 2 min and then cooled to 10°C. Sequenase dilution buffer and Sequenase enzyme were added and the sample was ramped up from 10°C to 37°C at 0.1°C/s, held at 37°C for 8 min and then cooled to 4°C. Samples were diluted and combined with a mix of *pfu* buffer, dNTP mix, Rand universal primer, and *pfu* polymerase. The DNA was cycled to 94°C for 30 s, 40°C for 30 s, 50°C for 30 s, and 72°C for 2 min for 25 cycles. The amplified DNA was purified using the Qiagen PCR purification kit according to protocol. The purified DNA was precipitated using 3M sodium acetate and ethanol overnight. Samples were centrifuged at 4°C for 1 h and subsequently washed with 70% ethanol, dried and resuspended in water.

Libraries for sequence analysis on the Illumina HiSeq 2000 using V3 chemistry were generated per the Illumina TruSeq standard protocol.

### Peak calling

HiSeq fastq files were generated and loaded into the CLC Genomics Workbench 7 software (CLCBio, Boston MA) for ChIP peak identification. Imported reads were mapped to the reference genome retrieved from NCBI. Default mismatch rates were applied, with length and similarity fractions both at 90%. All experimental samples were compared to the reference controls to identify binding events of interest. The most statistically significant peak calls at each time point were then visually verified using the GenomeView genome browser [19].

### Quantitative RT-PCR analysis

Total RNA was isolated by RNeasy mini kit, combined with RNA-free DNase for on-column DNase digestion (Qiagen) according to manufacturer’s instructions. cDNA was synthesized using the iScript cDNA synthesis kit (Bio Rad). The amount of cDNA corresponding to 10 ng of input RNA was used as template for real-time reaction containing Power SYBR green (Applied Biosystems) and gene-specific primers. The primers were designed with Primer Express 3.0 software and tested for amplification efficiencies (S2 Table). The *gyrB* gene, encoding for the B subunit of the DNA gyrase, was utilized as endogenous control. The RT-PCR reactions were carried out at 95°C for 10 min, 95°C for 15 s and 60°C for 1 min for 40 cycles (ABI 7700, Applied Biosystems). The expression ratio of each gene was the average from three independent RNA samples and was normalized to the level of *gyrB*.

## Results

### Identification of σ^E^-binding sites using ChIP-seq

We recently characterized the set of genes affected by σ^E^ in STM 14028s by microarray and identified 2533 genes exhibiting σ^E^-dependent transcription during growth in nutrient-rich and acidic minimal media. However, only 81 genes (3%) were regulated by σ^E^ in all of the growth conditions examined, suggesting genes regulated by σ^E^ were tuned by growth conditions that activated σ^E^ via different environmental cues [10]. In order to study the genomic binding sites of σ^E^ under conditions approximating the phagosomal environment, we infected murine macrophage Raw264.7 with STM and performed ChIP-seq on intracellular bacteria to identify genes directly bound by σ^E^ on the STM genome. To avoid the possible influence of epitope tagging on protein function, we generated primary anti-σ^E^ Ab, purified with affinity chromatography, and assessed its specificity by Western blot before applying it to ChIP. A single band at the correct size of σ^E^ (22 kDa) was detected in the cell lysate of the WT strain and absent in the Δ*rpoE* using anti-σ^E^ Ab (S1 Fig). When diluted 1:5000, the anti-σ^E^ Ab yielded a single reactive band. This evidence indicated that the generated anti-σ^E^ Ab was highly specific and had strong affinity. The monoclonal anti-GFP Ab, which did not show any cross reaction with STM, was used as negative control in the ChIP-seq experiments (S1 Fig).

**Fig 1:**
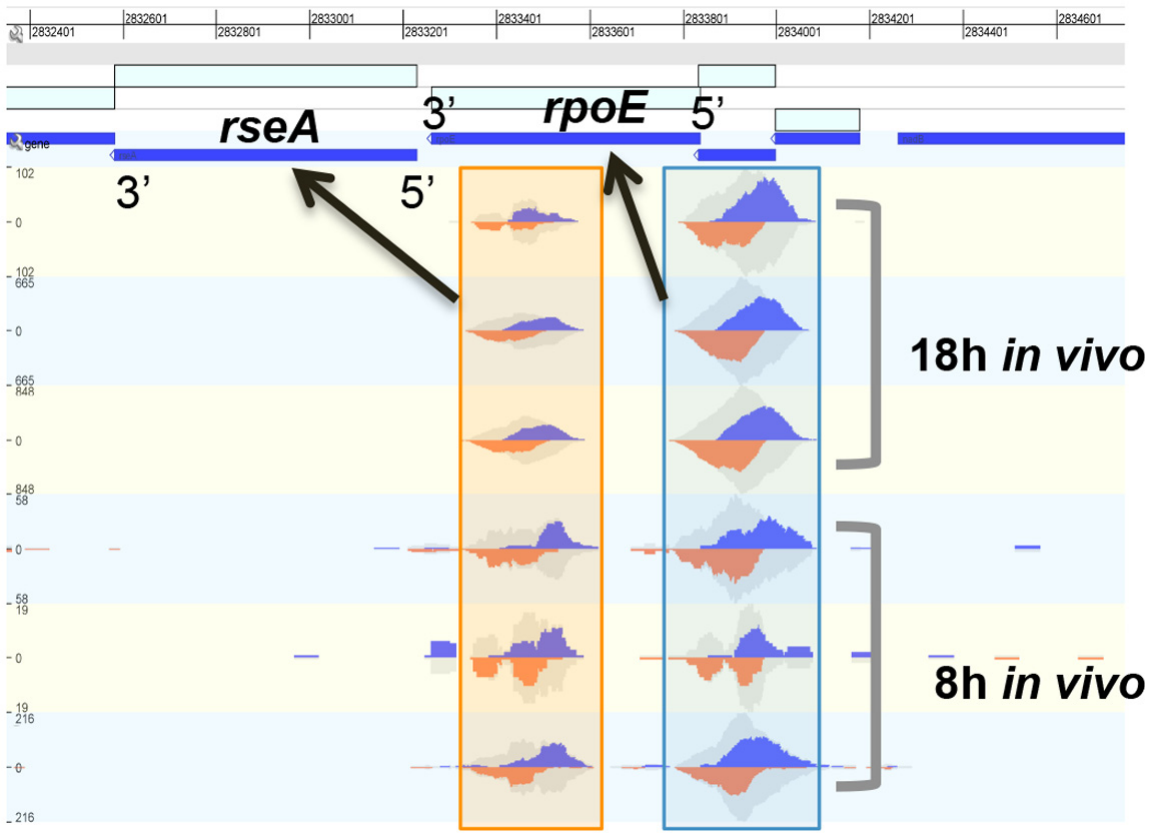
Visualization of the candidate binding sites (peak calls) upstream of *rpoE* and *rseA*. The peak calls for each replicate 8 h and 18 h are shown. The peaks in the blue box correspond to the binding site of σ^E^ to 5’ region of the gene *rpoE*, while those in the orange box correspond to the binding site 5’ to the gene *rseA*.

After peaks were called, the most statistically significant from each time point were visually verified. To pass this step, the read counts composing a peak were required to be substantially higher than background and also needed to be distributed evenly on both sides of the transcript (Fig 1). The peaks were ambiguous for the *in vivo* 4 h infection. We believe that the signal at this early time was not strong enough to provide robust results, so we excluded it from the analysis. A total of 31 ChIP-enriched peaks were identified for the 8 h and 18 h infections. Fourteen of the peak-associated genes were previously shown to be directly regulated by σ^E^ [8,9], supporting the effectiveness of the ChIP-seq procedure (Table 1). The remaining 17 genes represented new targets of varying function; encoding outer membrane proteins (*ompA*, *ompF*, and *ompN*), a quorum sensing protein (*luxS*) [20], a cell division factor (*minD*/*minE*) [21], a signal transduction modulator (*sixA*) [22], and hypothetical proteins (*stm14_1163*, *stm14_1514*, *stm14_1018*, *stm14_2321*, *stm14_2952*).

**Table 1:** σ^E^ ChIP peak-associated genes.

Chromosomal locus	Length	Possible Regulon Genes	Protein description
+(693332..693387)	55	*cspE*	cold shock protein CspE
+(3482416..3482496)	80	*dacB*; *greA*	D-alanyl-D-alanine carboxypeptidase; transcription elongation factor
+(3619604..3619693)	89	*fkpA*; *slyX*	FKBP-type peptidyl-prolyl cis-trans isomerase; hypothetical protein
+(2232101..2232161)	60	*galF*	UTP—glucose-1-phosphate uridylyltransferase subunit
+(245113..245172)	59	*htrA*	serine endoprotease
+(2987050..2987123)	73	*luxS*	S-ribosylhomocysteinase
+(1923425..1923495)	70	*minD*; *minE*	cell division inhibitor; cell division topological specificity factor
+(1120705..1120769)	64	*ompA*	outer membrane protein (Omp) A
+(1049412..1049491)	79	*ompF*; STM14_1131	OmpF precursor; hypothetical protein
+(1560795..1560885)	90	*ompN*; STM14_1778	OmpN precursor; hypothetical protein
+(900466..900535)	69	*ompX*; *ybiF*; STM14_969	OmpX; putative membrane protein
+(2659436..2659499)	63	*perM*; STM14_3058	putative permease; hypothetical protein
+(3393528..3393577)	49	*rpoD*	RNA polymerase sigma factor RpoD
+(2833864..2833944)	80	*rpoE*	RNA polymerase sigma factor RpoE
+(3750534..3750606)	72	*rpoH*	RNA polymerase factor sigma-32
+(2833414..2833514)	101	*rseA*	antisigma factor
+(4222610..4222683)	73	*spf*; STM14_4811	spot 42 RNA; hypothetical protein
+(2549605..2549664)	59	*sixA*	phosphohistidine phosphatase
+(943664..943722)	58	STM14_1018; *ybjM*	putative transport protein/regulator; putative inner membrane protein
+(1070680..1070759)	79	STM14_1163; STM14_1164	hypothetical protein; hypothetical protein
+(1348658..1348744)	86	STM14_1514; STM14_1515	hypothetical protein; putative ABC transporter binding protein
+(2012625..2012683)	58	STM14_2321	hypothetical protein
+(2565930..2565994)	64	STM14_2952; *ddg*	hypothetical protein; lipid A biosynthesis palmitoleoyl acyltransferase
+(107088..107150)	62	*surA*	peptidyl-prolyl cis-trans isomerase SurA
+(880214..880293)	79	*ybhP*; *ybhQ*	putative cytoplasmic protein; putative inner membrane protein
+(1045188..1045261)	73	*ycbK*	putative outer membrane protein
+(1891576..1891642)	66	*ychH*; *pth*	hypothetical protein; peptidyl-tRNA hydrolase
+(2860337..2860436)	99	*yfiO*; *yfiH*	OMP assembly complex subunit YfiO; hypothetical protein
+(3286581..3286626)	45	*yggN*	hypothetical protein
+(3386590..3386657)	67	*ygiF*; *ygiM*	putative cytoplasmic protein; putative signal transduction protein
+(3803794..3803859)	65	*yhjJ*	putative Zn-dependent peptidase

### Validation of *in vivo* σ^E^-binding sites

**Fig 2:**
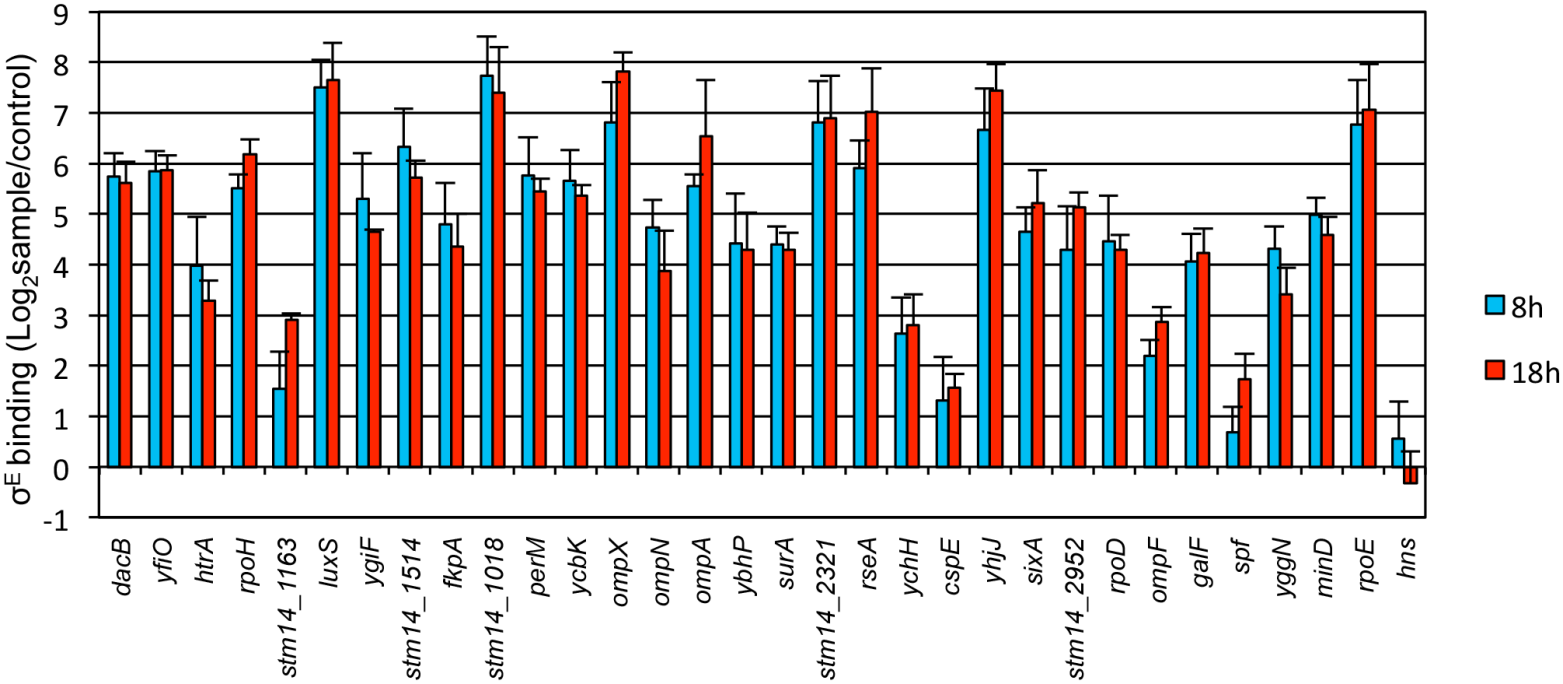
Validation of σ^E^ binding sites obtained with ChIP-seq. Raw264.7 cells were infected with WT bacteria for 8 h or 18 h, crosslinked, lysed, and sonicated for immunoprecipitation with anti-σ^E^ Ab (sample) or anti-GFP Ab (control). After protein and RNA were removed, DNA was extracted as template for qPCR to determine the fold enrichment of the genomic binding loci of σ^E^ in pulldowns with anti-σ^E^ Ab versus anti-GFP Ab. The promoter 3 region of *rpoE* was used as positive control, and the promoter region of *hns* was chosen as negative control. Shown are the fold changes displayed in a logarithmic scale. The mean and S.D. values were obtained from independent biological triplicates.

To validate σ^E^-binding, we performed ChIP-qPCR on target genomic loci and determined their fold enrichment in pulldowns using anti-σ^E^ Ab versus anti-GFP Ab (control). A known σ^E^–binding site, *rpoE* promoter 3, was pulled down in our ChIP-seq assay and used as positive control, and a non-target site, the promoter region of *hns*, was chosen as negative control [10]. For the 8 h infection, except for the binding site upstream of *spf*, all the ChIP-seq peaks exhibited more than 2-fold enrichment when compared to control samples, and were therefore considered authentic σ^E^-binding sites (Fig 2). For the 18 h infection, all of the ChIP-seq peaks were validated. For both conditions, binding sites upstream of *luxS*, *stm14_1018*, *ompX*, *stm14_2321*, and *yhjJ* exhibited enrichment as high as the positive control (*rpoE*, 64-fold), hence were considered high-affinity binding sites for σ^E^ (Fig 2).

### Identification of the consensus sequence of σ^E^-binding *in vivo*

**Fig 3:**
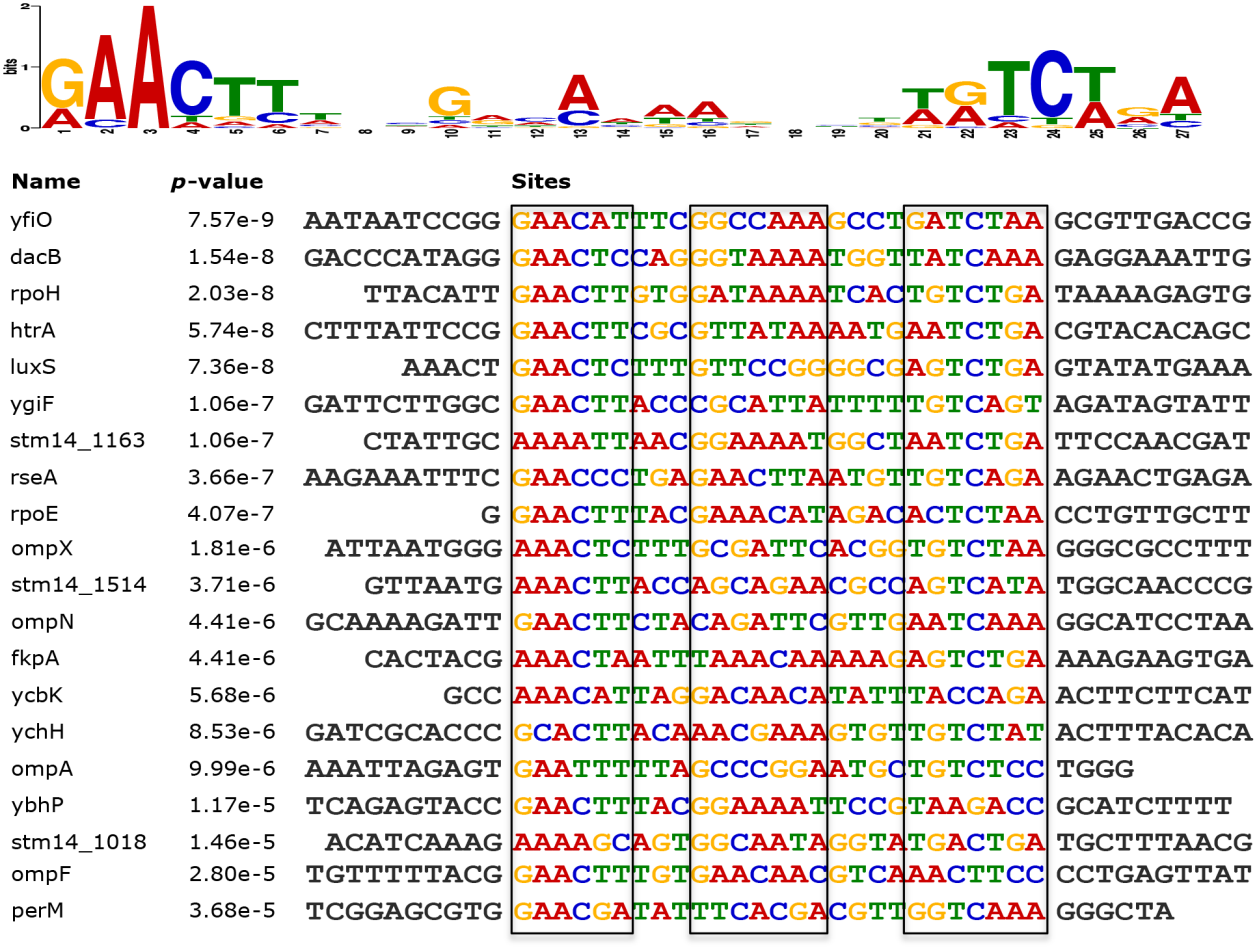
Consensus sequence of STM σ^E^ during *in vivo* infection. The top represents the sequence logos reflecting the prevalence of each symbol by height. Beneath is the nucleotide alignment of the ChIP-seq pulldowns that contain the consensus sequence (boxed).

The sequences of the 31 binding regions determined by our ChIP-seq experiments were analyzed with the MEME suite tool to identify a consensus sequence for σ^E^-binding [23]. For 20 of these peak regions, a consensus motif was identified (*p*-value < 0.0001). This A-T rich σ^E^ consensus sequence is largely similar to the one that was identified previously for STM [9], except that the nucleotides between the -35 and -10 regions appeared to contain conserved G and A in our *in vivo* σ^E^–binding consensus sequence. Except for the six genes (*rpoE*, *rpoH*, *fkpA*, *htrA*, *yfiO*, and *ygiM*) that were previously identified to contain σ^E^-dependent promoters [9], the σ^E^ consensus sequence was also found upstream of genes encoding outer membrane proteins (*ompA*, *ompF*, *ompN*, and *ompX*), putative permease (*perM*), putative cytoplasmic protein (*ybhP*) [24], transcription elongation factor (*greA*) [25], and quorum sensing autoinducer 2 synthase (*luxS*) [20] (Fig 3).

There were 11 σ^E^-binding sites (*cspE*, *galF*, *minD*, *rpoD*, *sixA*, *spf*, *stm14_2321*, *stm14_2952*, *surA*, *yggN*, *yhjJ*) validated in our study that did not contain any recognizable consensus sequence. However, some of these sites exhibited high σ^E^-binding capacity, such as *stm14_2321*, *ybjJ* (> 64-fold enrichment), and *stm14_1514*, *surA*, *sixA*, *stm14_2952*, *galF*, *minD* (> 16-fold enrichment) (Fig 2). This observation suggested that, besides the major regulatory mechanism of using the canonical consensus sequence, σ^E^ may utilize additional complementary mechanisms to regulate gene transcription.

### Establishing *in vitro* condition to mimic the *in vivo* σ^E^-binding

**Fig 4:**
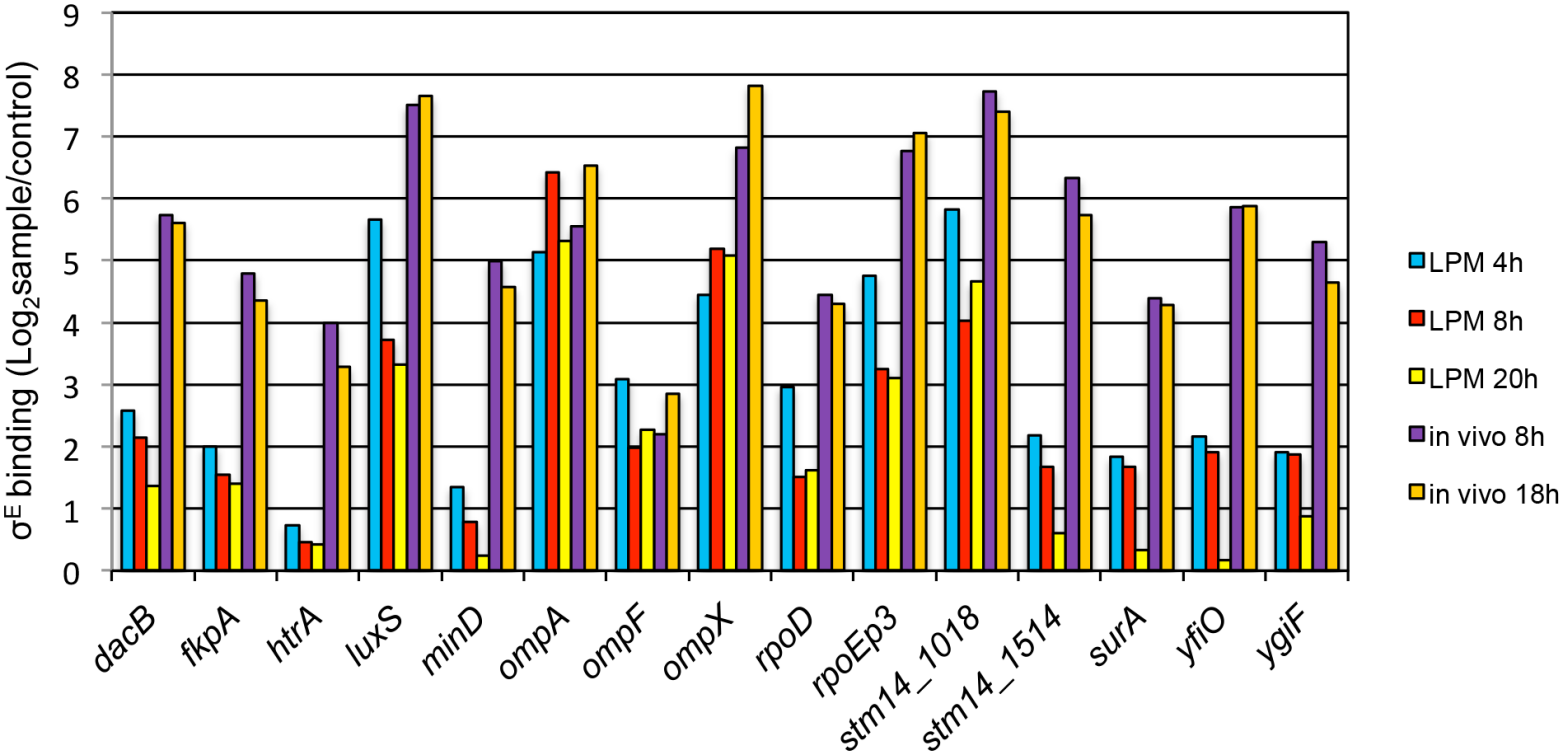
Comparison of σ^E^-binding sites between *in vitro* cultured conditions and *in vivo* infection. WT bacteria were cultured in acidic minimal medium (LPM) for 4 h, 8 h, or 20 h. ChIP and qPCR followed the same procedures as described in Fig 2. The fold enrichment of σ^E^-binding sites was determined in pulldowns with anti-σ^E^ Ab (sample) versus anti-GFP Ab (control). The *in vivo* infection results were imported from Fig 2.

The transcriptional profile of σ^E^ for STM intracellular infection is elusive because the *rpoE* mutation is so deleterious that the mutant can survive for no more than 30 min post-infection [5]. Therefore, we sought an *in vitro* method that could reproduce *in vivo* σ^E^-binding conditions. STM grown in LPM was used for mimicking *in vivo* infection [26], here the bacteria were cultured in LPM for 4, 8, and 18 h and then subjected to ChIP-qPCR as performed on *in vivo* samples. σ^E^-binding was evaluated on 15 sites selected from the *in vivo* ChIP-seq results (Fig 4). None of the three *in vitro* conditions could achieve similar binding capacity of σ^E^ on these sites as was seen in the *in vivo* conditions. However the LPM 4 h condition produced the most favorable result among them, where except for *htrA*, remaining binding sites were detected at levels higher than the 2-fold threshold. Hence, we used the LPM 4 h condition to study σ^E^-mediated gene regulation in an effort to approximate *in vivo* binding conditions.

### Dissecting direct versus indirect regulation mediated by σ^E^

**Fig 5:**
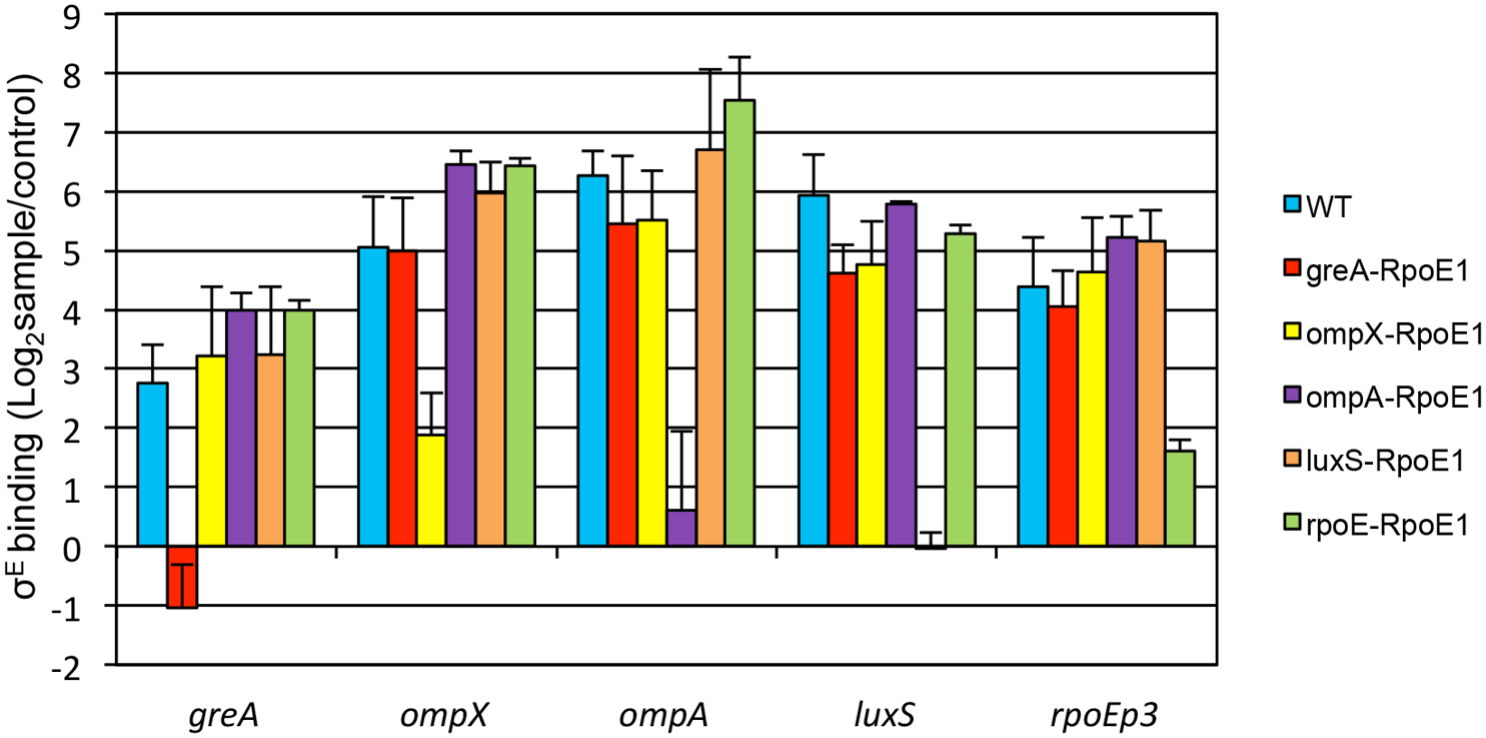
Scrambled σ^E^ consensus sequence decreases/eliminates σ^E^ binding. The substitutive consensus sequence consisted of nucleotides with the least prevalence at the corresponding positions in the consensus sequence. The σ^E^ consensus sequences upstream *greA*, *ompX*, *ompA*, *luxS*, and *rpoE* genes were replaced by the substitutive sequence (RpoE1), and the resulting mutants were named *greA*-RpoE1, *ompX*-RpoE1, *ompA*-RpoE1, *luxS*-RpoE1, and *rpoE*-RpoE1 respectively. These mutants and WT bacteria were grown in LPM for 4 h, crosslinked, sonicated, and immunoprecipitated with anti-σ^E^ Ab (sample) or anti-GFP Ab (control). qPCR was used to determine the binding of σ^E^ at each original site. The mean and S.D. values were obtained from independent biological triplicates.

Although 31 σ^E^-binding sites were recognized as directly regulated by σ^E^ during *in vivo* infection (Table 1, Fig 2), microarray was not sufficient to evaluate the regulatory effects of σ^E^ on these genes through comparing WT and Δ*rpoE* because this comparison measured total regulation mediated by σ^E^. Indirect regulation mediated by σ^E^, largely through controlled expression of multiple general regulators, accounted for the majority of the total regulation observed [10]. To dissect direct from indirect σ^E^–mediated regulation for *greA*, *ompX*, *ompA*, *luxS*, and *rpoE*, we replaced the authentic consensus sequence upstream of these genes with a scrambled consensus sequence, which resulted in 5 mutants designated greA-RpoE1, ompX-RpoE1, ompA-RpoE1, luxS-RpoE1, and rpoE-RpoE1. Mutant strains were cultured in LPM for 4 h and the σ^E^ binding capacity on 5 designated binding sites (*greA*, *ompX*, *ompA*, *luxS*, and *rpoE* promoter region) was examined using ChIP-qPCR (Fig 5). As expected, mutation of the σ^E^-binding motif upstream of each gene only eliminated (*greA*, *ompA*, *luxS*) or decreased (*ompX*) σ^E^ binding of each mutant to its corresponding sites, while leaving the other sites intact for σ^E^-binding. These results further confirmed the direct occupation of σ^E^ on these selected σ^E^-binding motifs.

**Fig 6:**
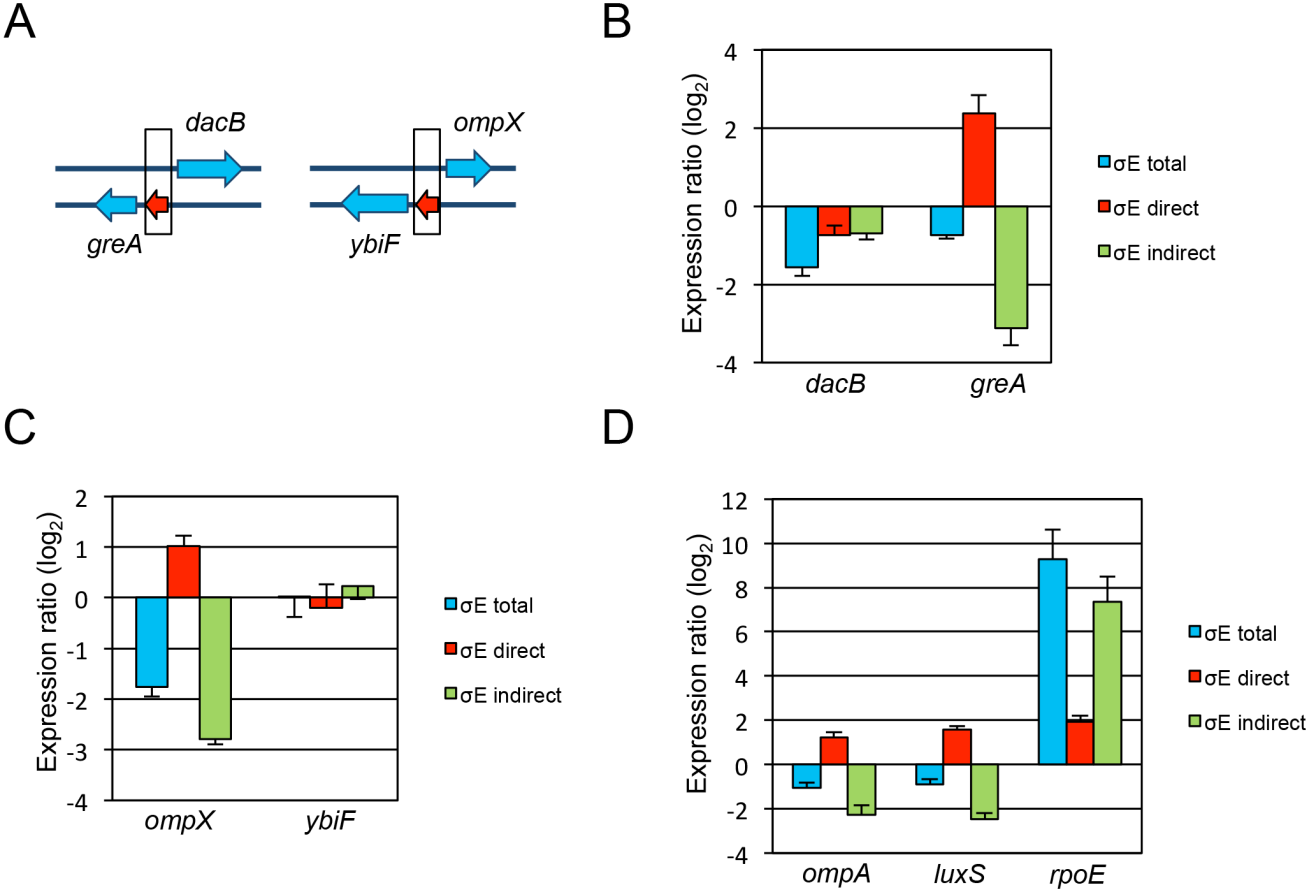
Dissection of the direct versus indirect regulatory effects of σ^E^ on gene expression. The total regulatory effect of σ^E^ on gene expression (σ^E^ total) was calculated by comparing the expression ratio of certain gene in WT versus Δ*rpoE* background (log_2_WT/*ΔrpoE*). The effect of σ^E^ as a result of direct binding to the 5’ element of a particular gene (σ^E^ direct) was calculated by comparing the expression ratio of the gene in WT versus its σ^E^ consensus sequence substituted mutant. The indirect regulatory effect of σ^E^ (σ^E^ indirect) was calculated by comparing the expression ratio of the gene in Δ*rpoE* versus its σ^E^ consensus sequence substituted mutant. (A) Diagram of σ^E^ consensus sequence (red) in relation to bi-directional flanking genes (*greA* versus *dacB*; *ybiF* versus *ompX*). The black rectangle represents σ^E^–binding site identified by ChIP-seq. (B) σ^E^-binding in between *dacB* and *greA* genes directly activated *greA* instead of *dacB*. (C) σ^E^-binding in between *ompX* and *ybiF* genes directly activated *ompX* instead of *ybiF*. (D) σ^E^ directly activated *ompA*, *luxS*, and *rpoE* expression.

Of the 31 σ^E^-binding sites identified, 16 of them were located between bi-directional promoter elements (Table 1). Since the σ^E^-binding site was 5’ to genes in both directions, we further investigated if σ^E^ regulated these genes equally. Two σ^E^-binding sites were chosen, one located between *dacB* and *greA*, with a second between *ompX* and *ybiF* (Fig 6A). WT, Δ*rpoE*, and consensus sequence substituted mutants (*greA*-RpoE1 or *ompX*-RpoE1) were cultivated in LPM for 4 h, and expression of the genes in question was measured using qRT-PCR with *gyrB* serving as the internal control. The total regulatory effects of σ^E^ on a particular gene was calculated by comparing its expression ratio in WT versus Δ*rpoE*. The direct contribution of σ^E^ was measured by the expression ratio in WT versus the consensus sequence substituted mutant, and the indirect contribution of σ^E^ was measured by the expression ratio in Δ*rpoE* versus the consensus sequence substituted mutant. We found that based on total regulation, it was apparent that σ^E^ regulated *dacB* instead of *greA*. However the indirect regulation of σ^E^ on *greA* was much higher than that on *dacB*, leading to the net regulation directly mediated by σ^E^ significant on *greA*, but not *dacB* (Fig 6B). Similarly, dissecting σ^E^ regulation indicated that σ^E^ activated *ompX* instead of *ybiF* (Fig 6C). Three more σ^E^-binding sites (5’ to *ompA*, *luxS*, and *rpoE*) were examined. σ^E^ activated each locus through direct occupation of their σ^E^ consensus sequence (Fig 6D). Moreover, the direct effect of σ^E^ in regulating its own expression (*rpoE*) was much lower than the total and the indirect contributions of σ^E^. The dissection of σ^E^-mediated regulation clarified that σ^E^ activated gene expression by binding to the consensus sequence (such as *greA*) or reverse consensus sequence (such as *ompX*). The previous findings of σ^E^ repressing gene expression by comparing the level of expression in WT and Δ*rpoE* was therefore misleading, in that indirect regulation of σ^E^ expression itself played a large role in defining activation versus repression. When the indirect regulation of σ^E^ is subtracted from the total regulation mediated by σ^E^, the resulting direct regulation of σ^E^ is activation of gene expression. Together, our results clearly elucidated σ^E^-mediated regulation by dissecting direct versus indirect regulation.

### Genes regulated by σ^E^ are involved in heat shock and oxidative stress response

**Fig 7:**
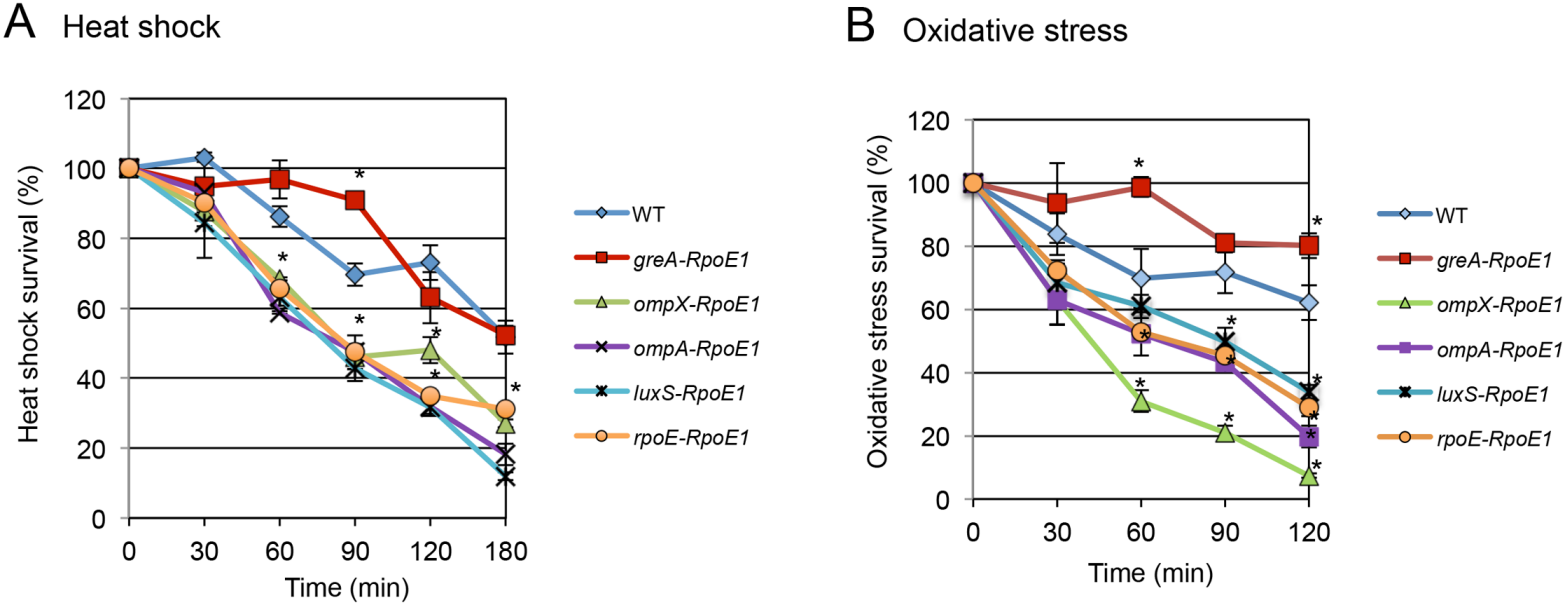
Heat shock and oxidative stress susceptibility. WT and mutant strains with substituted σ^E^ consensus sequences (refer to Fig 5. for details) were cultured in LB broth overnight, washed with and re-suspended in M9 minimal medium before challenging with heat shock (48°C) (A) or oxidative stress (5 mM H_2_O_2_) (B) The mean and S.D. values were obtained from three independent experiments.

The alternative sigma factor σ^E^ is activated under extracytoplasmic stress such as heat shock, ethanol, osmotic stress, immune response etc. [4]. By regulating gene expression at both the transcriptional and post-transcriptional levels [11], σ^E^ functions to maintain the integrity and homeostasis of the cell. We further investigated if newly identified targets of σ^E^-binding served to fulfill this purpose. The WT and consensus sequence substituted mutants (*greA*-RpoE1, *ompX*-RpoE1, *ompA*-RpoE1, *luxS*-RpoE1, and *rpoE*-RpoE1) were challenged by heat shock and oxidative stress (Fig 7). Compared to WT, the susceptibility of all mutants to heat shock was significantly altered at 90 min post-challenge. Except for *greA*-RpoE1, all other mutants exhibited lower resistance to heat shock. At the 3 h time point, the *greA*-RpoE1 strain recovered to WT levels, whereas the remaining mutants were significantly hyper-sensitive to heat shock when compared to WT (Fig 7A). Under oxidative stress, the mutant strains exhibited higher (*greA*-RpoE1) or lower (*rpoE*-RpoE1 and *ompX*-RpoE1) susceptibility than WT at 60 min post-challenge. At the 2 h time point, all mutant strains showed significantly altered sensitivity to oxidative stress compared to WT (Fig 7B). These results suggested the genes regulated by σ^E^ (*greA*, *luxS*, *ompA*, and *ompX*) were involved in heat shock and oxidative stress response.

## Discussion

The transcriptional profile of σ^E^ has been studied using microarray by comparing WT to Δ*rpoE* strain or by comparing WT to *rpoE*-overexpressed strain [10] [8]. Since microarray cannot distinguish direct from indirect regulation, a large amount of genes were found to be regulated by σ^E^, and the number of genes activated by σ^E^ was similar to those repressed [10]. Although the *Salmonella* σ^E^-regulon associated with direct binding has been studied using an *E*. *coli* two-plasmid screening system [9], the genome-wide identification of σ^E^-binding sites during *Salmonella in vivo* infection has not been investigated. The *in vivo* σ^E^-binding sites are likely different from *in vitro* because the signals perceived by σ^E^ from these two environments are different. Thirty-one σ^E^-binding sites were identified during *Salmonella in vivo* infection in this study, and when compared with *in vitro* culture on fifteen of these sites, the binding capacity of σ^E^ in LPM 4, 8, and 20 h culture was lower on the majority of the sites measured (Fig 4). How σ^E^ selectively binds to some sites but not other potential sites under certain conditions is not known. However, other general regulators such as H-NS, IHF, and Fis might play a role in affecting the direct binding of σ^E^ to these sites [14]. Out of the thirty-one *in vivo* σ^E^-binding sites, sixteen were located upstream of bi-directional promoter elements (Table 1). We found that those flanking genes were not equally regulated by σ^E^ under the conditions examined (Fig 6).

The σ^E^ consensus sequence predicted here during *Salmonella* infection was similar to the consensus sequence identified by the *E*. *coli* two-plasmid system [9], and also comparable to the consensus sequence identified in *E*. *coli* [8], suggesting conservation of sequence features related to σ^E^-binding. Both this and previous studies found that the σ^E^ consensus sequence located upstream of *ompX* was reversed, and the microarray data indicated that σ^E^ represses *ompX* expression [8] [10]. Since *ompX* was found to be directly regulated by σ^E^, the repression effect of σ^E^ on outer membrane proteins was hypothesized as a conserved feature [8]. We constructed a consensus sequence substituted strain and by comparing with WT and *ΔrpoE* strains, successfully dissected the direct versus indirect contribution of σ^E^. The results clearly demonstrated that σ^E^ directly activated *ompX* expression through the reversed consensus sequence. The perception of σ^E^-mediated repression was due to the high-level of indirect effects of σ^E^. Except for *ompX*, other genes encoding outer membrane proteins (*ompA*, *ompF*, and *ompN*) were additionally directly regulated by σ^E^. Similar to *ompX*, the total effect of σ^E^ on the expression of these genes is not activation [10], likely mediated by σ^E^–dependent small RNAs that accelerate global *omp* mRNA decay upon membrane stress [27].

We found that a considerable amount of σ^E^-binding sites were associated with genes involved in transcriptional circuitry (*rpoE*, *rpoH*, *rpoD*, *rseA*, *greA*, and *sixA*) [25] [22], protein folding (*fkpA*, *surA*, and *htrA*) [28] [29] [30], protein biosynthesis (*pth*) [31] and assembly (*yfiO*) [32], stress adaptation (*cspE*, *galF*, and *luxS*) [33] [34,35], cell division (*minD*/*minE*) [21], and lipid A biosynthesis (*ddg*) [36]. Upon activation of σ^E^, a positive feedback on itself ensures sufficient σ^E^ is produced to accelerate the protein synthesis required for regaining homeostasis. As a strategy to avoid imbalances, σ^E^ also regulates the anti-sigma factor *rseA*, which dampens the σ^E^ response as a negative feedback loop [8]. Other alternative factors are also activated by σ^E^, not only because some of the stresses they respond to are overlapping, but also to expand the protection to other stresses the bacteria may encounter in later stages [37]. A previous study identified a cell division protein FtsZ is regulated by σ^E^ in eight organisms closely related to *E*. *coli* [8]. Here, we found σ^E^ regulates other cell division factors (*minD*/*minE*), further suggesting that σ^E^ may orchestrate factors associated with cell division. Both outer membrane proteins (OmpA, OmpF, OmpN, and OmpX) and proteins promoting OMP assembly (FkpA, HtrA, and YfiO) are regulated by σ^E^, hence facilitating the proper assembly and insertion of the newly synthesized OMPs into the outer membrane of *Salmonella*.

Activation of σ^E^ is thought to maintain bacterial homeostasis under extracytoplasmic stresses. Five σ^E^-binding sites (*greA*, *ompX*, *ompA*, *luxS*, and *rpoE*) were tested for the fulfillment of this purpose using heat shock and oxidative stress challenges (Fig 7). All of them exhibited significantly altered susceptibility to these stresses, consistent with the traditional role of σ^E^. Moreover, this finding supported the observation that the transcription elongation factor GreA has functional chaperone activity [38], which is likely regulated by σ^E^. Why the quorum sensing protein LuxS is involved in extracytoplasmic stress response needs to be further investigated. The σ^E^ regulatory system is flexible and efficient, hence playing a significant role for *Salmonella* to survive in various environments.

## Supporting Information

S1 Fig The quality of antibodies for ChIP experiments. The rabbit Ab to σ^E^ and monoclonal Ab to GFP were tested for specificity by western blot. Cell lysates from WT or *ΔrpoE* strains were loaded on the gel, and different Abs were used to develop the membrane. Lane 1, the flow through of rabbit immune sera during affinity chromatography purification of anti-σ^E^ Ab. Lane 2–4, Ab to σ^E^ diluted at 1:1000, 1:3000, and 1:5000 respectively. Lane 5–7, monoclonal Ab to GFP diluted at 1:1000, 1:3000, and 1:5000 respectively.
(TIF)

S1 Table List of primers used for mutant construction. (XLSX)

S2 Table List of quantitative PCR primers used in this study. (XLSX)
