# Supplementary figures and images for: ChIP-Seq Analysis of the σE Regulon of Salmonella enterica Serovar Typhimurium Reveals New Genes Implicated in Heat Shock and Oxidative Stress Response
Source: PLoS One. 2015 Sep 21;10(9):e0138466. doi: 10.1371/journal.pone.0138466 (PMC4577112; doi:10.1371/journal.pone.0138466)

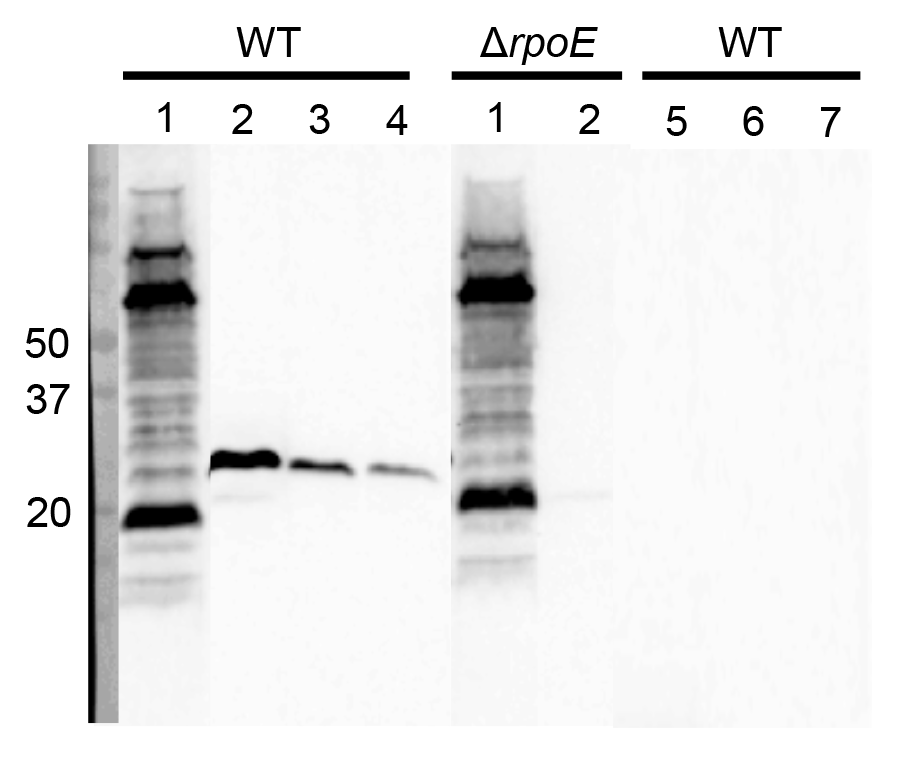

Supplement: S1 Fig — The rabbit Ab to σE and monoclonal Ab to GFP were tested for specificity by western blot. Cell lysates from WT or ΔrpoE strains were loaded on the gel, and different Abs were used to develop the membrane. Lane 1, the flow through of rabbit immune sera during affinity chromatography purification of anti-σE Ab. Lane 2–4, Ab to σE diluted at 1:1000, 1:3000, and 1:5000 respectively. Lane 5–7, monoclonal Ab to GFP diluted at 1:1000, 1:3000, and 1:5000 respectively. (TIF) [file pone.0138466.s001.tif]
